# Perception of the duration of emotional faces in schizophrenic patients

**DOI:** 10.1038/srep22280

**Published:** 2016-02-29

**Authors:** Dandan Zhang, Yanli Zhao, Yunzhe Liu, Shuping Tan

**Affiliations:** 1 Institute of Affective and Social Neuroscience, Shenzhen University, Shenzhen 518060, China; 2 Center for Psychiatric Research, Beijing Huilongguan Hospital, Beijing 100096, China

## Abstract

The level of emotional timing deficit is a critical determinant of daily functions and social interactions in people with schizophrenia. This study demonstrated that people with schizophrenia have significant deficits in emotional time perception. Behaviorally, while the healthy controls overestimated the duration of happy and fearful faces, the patients underestimated the duration of emotional and neutral faces. Accordingly, an online ERP index of timing—the contingent negative variation (CNV) displayed larger amplitudes for emotional faces in the controls, whereas the CNV in the patients only showed overall smaller amplitudes when compared with the controls. In addition, the results of the N170 and the CNV suggest that the emotional processing and timing for facial expressions in schizophrenia might have a pattern of two-stage deterioration. Findings from the present work point to the importance of considering the time dimension of emotional processing in schizophrenia, based on which we are likely to discover aspects of emotional deficits that would be unnoticed in other studies. Furthermore, the perception deviation of the duration of emotional faces in schizophrenia suggests us to consider the magnitude of this temporal deviation as a quantitative biomarker for specific emotional/social dysfunctions in schizophrenia.

The past two decades of research on emotional processing in schizophrenic populations has revealed that the impaired processing of emotion, which is evident at the onset of psychosis and persists during acutely symptomatic and remitted states, represents a trait susceptibility marker and a unique endophenotype for schizophrenia^1^,^2^. Compared to people without schizophrenia, patients with schizophrenia display impaired emotional perception, diminished emotional expression, and a relatively normal level of emotional experience^3^,^4^,^5^.

However, when considering the temporal course of emotion, the function of emotional processing in schizophrenic patients may display a more refined or even distinctively different pattern^5^. For instance, studies have demonstrated that the timing of emotional expression/experience is different between people with and without schizophrenia: people with schizophrenia are able to exhibit emotionally congruent, albeit less observable, expressions to facial pictures, but only during the first 500 ms of picture presentation^6^; people with and without schizophrenia show comparable regions of brain activation in the presence of emotional pictures, but patients cannot maintain their emotional experience responses after the offset of stimuli^7^. Furthermore, many researchers are currently stressing the importance of characterizing the temporal course of emotional experience to distinguish anticipatory from consummatory responses in schizophrenia, since accumulating evidences reveal that people with schizophrenia do not have a deficit in consummatory pleasure but instead have a deficit in anticipatory pleasure^8^,^9^,^10^; and that it is the deficit in anticipatory pleasure that results in a symptom of anhedonia^9^. (Note: consummatory pleasure is the pleasure being received when individuals are directly engaged in an enjoyable activity; anticipatory pleasure is the experience of pleasure related to future activities^9^).

Whereas these results consistently support the notion that people with schizophrenia have difficulty in anticipating emotional events and maintaining their emotional responses and experiences^5^, analysis of the complex interplay between emotion and timing remains relatively rare in this population. Impaired time perception of emotional stimuli, such as faces and voices, is associated with problems in synchronizing our activity with other individuals, adopting others’ rhythms, and understanding the intentions of others^11^. In addition, the core manifestations of schizophrenia such as thought disorder and contextually inappropriate responses may be partly due to the time perception dysfunction for emotional events/cues^12^. Thus, the level of emotional timing deficit appears to be a critical determinant of daily functioning and social interactions in schizophrenia.

The present study investigated and compared the behavioral and the event-related potential (ERP) responses of emotional time perception in individuals with and without schizophrenia. Since emotional facial expressions play an essential role in social communication, pictures of emotional faces were used as the target of time perception^11^. Accordingly, we investigated ERP components of P1, N170, the vertex positive potential (VPP), and the contingent negative variation (CNV) in this study. The P1 reflects bottom-up visual processing and is sensitive to attention and stimulus parameters^13^. The N170 and its positive counterpart VPP are face-sensitive components; both are frequently modulated by emotional faces, with larger amplitudes in response to emotional, compared to neutral, facial expressions^14^,^15^. The CNV is a slow negative potential which has been shown in numerous studies of time perception as an online index of timing^16^,^17^; its amplitude correlates positively with the length of the estimated duration^18^,^19^,^20^. It is expected that the emotion-sensitive N170/VPP would show different patterns across emotional conditions between schizophrenic patients and healthy controls; and that the timing-sensitive CNV displays not only group differences but also a significant interaction of emotion by group, reflecting that general time perception and emotion-modulated time perception are both impaired in individuals with schizophrenia.

This study also aims to investigate the potential relationship between the deficits in emotional time perception and the emotional/social impairments in schizophrenic patients. Clinically, the severity of emotional/social impairments is often assessed using the Positive and Negative Syndrome Scale (PANSS)^21^. An accumulating literature suggests that the severity of negative, rather than positive, symptoms, reflects poor performances on emotion-related tasks^4^,^22^ and severe social dysfunctions in schizophrenic patients^23^,^24^. Factor analytic studies suggest that negative symptoms consistently have two factors linked to emotional/social dysfunction^24^,^25^. Among the seven items of negative symptoms, three (“blunted affect”, “poor rapport”, and “lack of spontaneity and flow of conversation”) are grouped into the dimension of emotional expressive deficit (or diminished expression), and two (“emotional withdrawal” and “passive social withdrawal”) are grouped into the dimension of social amotivation (or anhedonia-asociality)^23^,^26^. Accordingly, these five negative items are our major concerns in this study. It is expected that the ERP abnormity found in the emotional time perception task would correlate with specific negative items in the PANSS.

## Methods

### Participants

Forty-seven inpatients (27 males) of Beijing Huilongguan Hospital and forty-six (26 males) normal controls were recruited as paid participants.

Patients were diagnosed according to the criteria for Schizophrenia in Diagnostic and Statistical Manual (DSM-IV)^27^. The diagnosis was based on structured clinical interview for DSM (SCID)^28^. Patients with schizoaffective disorder, schizotypal or schizoid personality disorder were excluded. None of the patients were in a major depressive or manic episode at the time of testing. Additional exclusion criteria included: 1) history of significant brain trauma, 2) neurological disorder, 3) substance abuse or dependence in the past six months, 4) IQ < 70, and 5) who had received electroconvulsive therapy in the past six months. All patients were receiving stable medication treatments (no medication changes) for at least one month before the experiment. Patients’ symptom severity was assessed using the PANSS within ten days of ERP assessment.

Healthy control participants were screened with the SCID-I/NP^29^ and SCID-II^30^. Exclusion criteria were 1) any lifetime Axis I psychotic or mood disorders, 2) recurrent depression, 3) paranoid, schizotypal or schizoid personality disorder, 4) seizure disorder, 5) history of head injury with possible neurological sequelae, 6) the presence of a first-degree relative with schizophrenia, and 7) substance abuse or dependence in the past six months.

There was no significant difference between the two groups with respect to age, handedness, IQ, education, and sex (Table 1). The interview and clinical symptom rating were based on consensus of two senior psychiatrists who were trained with a high reliability (κ = 0.83). The experimental protocol was approved by the local ethics committee (Beijing Huilongguan Hospital) and this study was performed strictly in accordance with the approved guidelines. Written informed consent was obtained from participants prior to the experiment.

### Stimuli and procedure

The stimuli used for the representation of duration were a grey oval and three types of facial pictures, namely, happy, fearful and neutral faces. Facial pictures were black and white photographs selected from the Chinese Facial Affective Picture System^31^, with equal number of facial pictures between males and females. A total of 72 faces were used (24 happy, 24 fearful and 24 neutral ones). All stimuli were presented with the same contrast and brightness on the black background (4.0° × 4.6° visual angle).

The 72 facial pictures had been assessed for its valence and arousal on a 9-point scale by another 100 Chinese adults; and the demographic data between the 100 adults and the control participants of this study were very similar (refer to^31^). The ANOVA performed on the average scores of the 72 faces showed that the three categories of faces have significantly different emotional valence scores (*F*(2,69) = 219, *p* < 0.001, 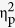 = 0.864; happy = 6.24 ± 0.12, fear = 2.70 ± 0.12, neutral = 4.20 ± 0.12) as well as arousal scores (*F*(2,69) = 51.6, *p* < 0.001, 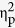 = 0.600; happy = 5.78 ± 0.21, fear = 6.58 ± 0.21, neutral = 3.64 ± 0.21).

**Figure 1:**
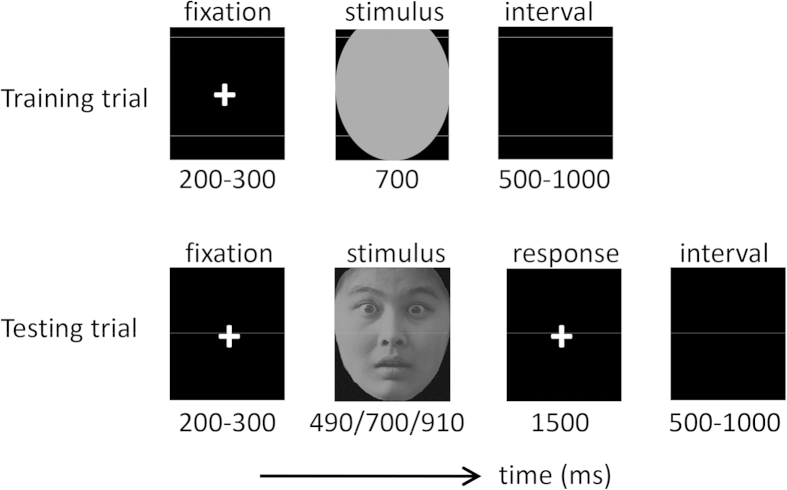
Illustration of one training trial and one testing trial in this study. In the training phase, the “standard” stimulus (a grey oval) was presented for 700 ms. In the testing phase, participants watched three durations (490, 700 and 910 ms) presented in the form of happy, fearful or neutral faces.

The temporal judgment task was composed of two phases-a training phase and a testing phase (Fig. 1). In the training phase, participants watched the “standard” stimulus duration (700 ms) ten times, represented by the grey oval. In the testing phase, participants watched three comparison durations (490, 700 and 910 ms)^19^,^32^ presented in the form of happy, fearful or neutral faces. Participants were required to judge whether the face was presented in shorter, longer or equal durations compared with the standard stimulus they had studied by pressing the “left”, “right” or “up” buttons, respectively, on the joystick with their right thumb.

The testing phase consisted of 9 blocks (each containing 48 testing trials). Each face was presented twice in each of the three duration conditions. The emotion category and the presentation duration of faces varied randomly across trials. The standard stimulus was presented five times at the beginning of each block to prevent the participants from forgetting it.

### EEG recording and analysis

Brain electrical activity was recorded referentially against left mastoid and off-line re-referenced to the average of bilateral mastoids, by a 64-channel amplifier with a sampling frequency of 250 Hz (Brain Products, Gilching, Germany). The recorded data were band-pass filtered online within 0.01–80 Hz. Ocular artifacts were removed from EEGs using a regression procedure implemented in NeuroScan software (Scan 4.3). The EEG data (after ocular artifacts removed) were filtered (0.01–30 Hz), segmented, and baseline-corrected (−200 to 0 ms), followed by averaging in association with experimental conditions irrespective of response^19^,^33^.

This study focused on the peak amplitudes and the peak latencies of P1, N170, and VPP, as well as the area amplitude of CNV across different sets of electrodes according to grand-mean ERP topographies and relevant literatures^13^,^14^,^19^. The CNV was estimated using an area measurement which was calculated based on the integral under the ERP waveforms between two zero crossing points on the time axis^20^. The measures of P1 were calculated as the mean value of the electrode sites of O1 and PO3 (for left hemisphere), and the mean value of O2 and PO4 (for right hemisphere), within a time window for peak detection of 90–150 ms. Similarly, the measures of N170 were calculated as the mean value of P7 and PO7 (for left hemisphere), and the mean value of P8 and PO8 (for right hemisphere), within a time window for peak detection of 140–200 ms. The components of VPP and CNV were measured irrespective of hemisphere, and were calculated as the mean value of FC1, FCz and FC2 (time window for VPP peak detection = 140–200 ms).

### Statistics

Descriptive data were presented as mean ± standard error. Repeated-measures ANOVA was performed on measurements of ERP and behavioral data, with emotion (happiness, fear, and neutral) as the within-subject factor, and group (patient and control) as the between-subject factor. When analyzing the P1 and the N170, another within-subject factor of hemisphere (left and right) was included. When analyzing the CNV and behavioral measures, the within-subject factor of stimulus duration (490, 700, and 910 ms) was added. In addition, the response count was compared between different conditions, with emotion, stimulus duration, and response (short, equal and long) as the within-subject factors, and group as the between-subject factor. Greenhouse-Geisser and Bonferroni corrections were used whenever appropriate. Two-tailed Pearson’s r correlation was performed between negative symptoms and the ERP measurements of patients (Holm’s stepwise correction).

## Results

For the sake of brevity, this section only reports the most important results. Please refer to the supplementary material for the other significant findings.

### Behaviors

#### Accuracy rate

The main effect of group was significant (*F*(1,91) = 98.8; *p* < 0.001; 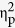 = 0.521). The ACC was lower in the patients (0.474 ± 0.013) than in the controls (0.656 ± 0.013).

#### The “short”, “equal”, and “long” responses

**Figure 2:**
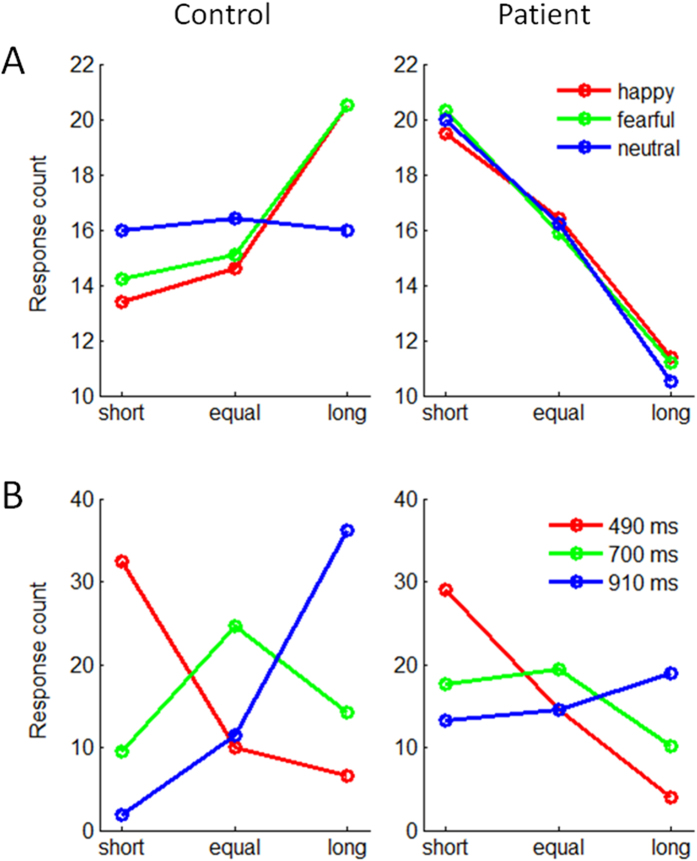
The behavioral results of the “short”, “equal”, and “long” responses. (**A**) the interaction effect of emotion by response by group; (**B**) the interaction effect of stimulus duration by response by group.

The interaction effect of emotion by response by group was significant (*F*(4,364) = 5.11; *p* = 0.001; 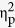 = 0.053; Fig. 2A). The controls tended to give more “long” responses (happy = 20.5 ± 1.14 counts; fearful = 20.5 ± 1.07 counts) compared with “equal” (happy = 14.6 ± 1.36 counts, *p* = 0.003; fearful = 15.1 ± 1.34 counts, *p* = 0.013) and “short” responses (happy = 13.4 ± 0.77 counts, *p* < 0.001; fearful = 14.2 ± 1.10 counts, *p* < 0.001) in the happy (*F*(2,184) = 7.30; *p* = 0.001) and the fearful conditions (*F*(2,184) = 5.12; *p* = 0.007), while they gave the three responses almost equally in the neutral condition (*F*(2,184) < 1). However, the patients consistently gave more “short” responses (happy = 19.5 ± 0.76 counts; fearful = 20.3 ± 1.09 counts; neutral = 20.0 ± 0.89 counts) compared with “long” responses (happy = 11.4 ± 1.13 counts; fearful = 11.2 ± 1.06 counts; neutral = 10.5 ± 1.10 counts; *p*s < 0.001) in the three emotional conditions (*F*(2,184) = 8.62 to 12.3; *p*s < 0.001). In addition, the interaction effect of stimulus duration by response by group was significant (Fig. 2B; refer to supplementary materials for details).

There were 48 trials in each experimental condition. Responses given within 1500 ms were considered valid data. The main effect of group was significant (*F*(1,91) = 28.0; *p* < 0.001; 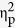 = 0.235). Compared with the controls (16.3 ± 0.08 counts), the patients gave less valid responses (15.7 ± 0.08 counts).

#### Response time

**Figure 3:**
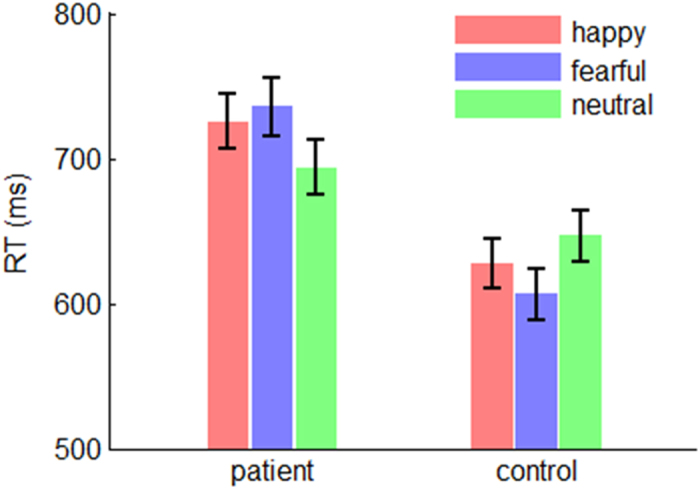
The interaction effect of emotion by group on the reaction time. Bars represent ± standard error of the mean.

The interaction effect of emotion by group was significant (*F*(2,182) = 9.20; *p* < 0.001; 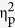 = 0.092; Fig. 3). The response of the patients (*F*(2,182) = 5.16; *p* = 0.007) was slower in the fearful condition (735 ± 14.4 ms) compared with that in the neutral condition (694 ± 15.3 ms; *p* = 0.008). In contrast, the response of the controls (*F*(2,182) = 4.28; *p* = 0.015) was quicker in the fearful condition (606 ± 11.0 ms) compared with that in the neutral condition (646 ± 12.8 ms; *p* = 0.003).

The main effect of group was significant (*F*(1,91) = 34.9; *p* < 0.001; 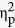 = 0.277). The response was slower in the patients (718 ± 10.9 ms) than in the controls (627 ± 11.0 ms).

### ERPs

#### The P1

##### Peak amplitude

**Figure 4:**
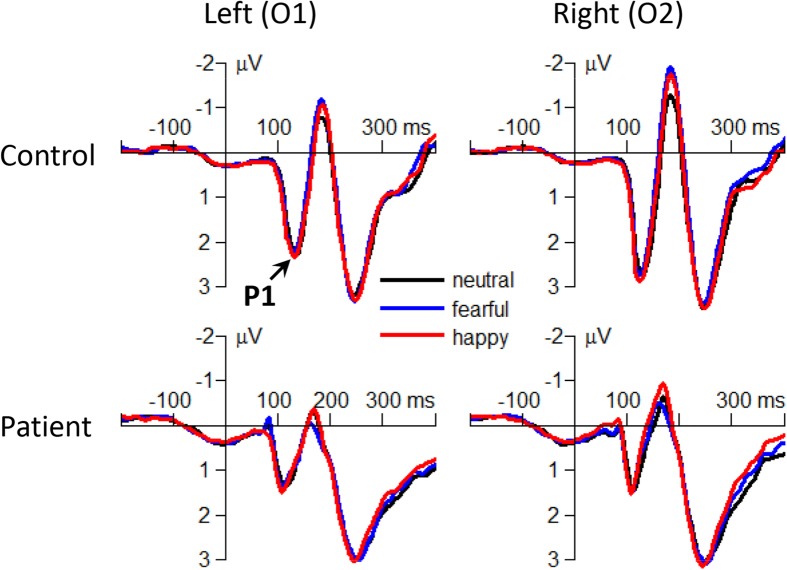
The grand-mean ERP waveforms of the P1 component in neutral, fearful and happy conditions in the patients and controls.

The main effect of group was significant (*F*(1,91) = 77.8; *p* < 0.001; 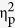 = 0.461) (Fig. 4). The P1 amplitude evoked in the patients (2.34 ± 0.09 μV) was smaller than that evoked in the controls (3.48 ± 0.09 μV).

#### The N170

##### Peak amplitude

**Figure 5:**
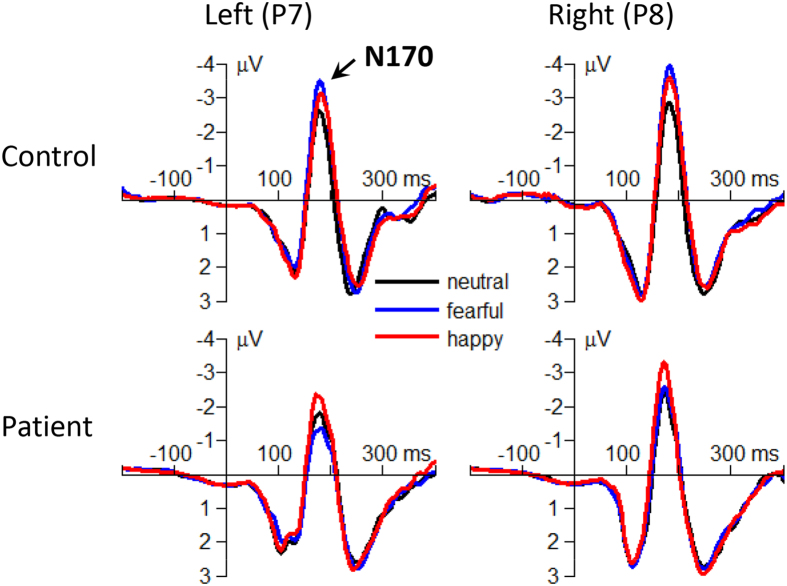
The grand-mean ERP waveforms of the N170 component in neutral, fearful and happy conditions in the patients and controls.

The interaction effect of emotion by group was significant (*F*(2,182) = 19.9; *p* < 0.001; 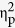 = 0.179; Fig. 5). The N170 amplitude evoked in the patients (*F*(2,182) = 15.2; *p* < 0.001) was larger in the happy condition (−3.76 ± 0.12 μV) compared with that in the fearful (−3.00 ± 0.11 μV; *p* < 0.001) and the neutral conditions (−3.26 ± 0.13 μV; *p* = 0.012). However, the emotion effect showed a different pattern in the controls (*F*(2,182) = 19.3; *p* < 0.001): the N170 amplitude was smaller in the neutral condition (−3.82 ± 0.13 μV) compared with that in the happy (−4.38 ± 0.13 μV; *p* < 0.001) and the fearful conditions (−4.68 ± 0.11 μV; *p* < 0.001).

The main effect of group was significant (*F*(1,91) = 56.6; *p* < 0.001; 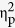 = 0.383). The N170 amplitude evoked in the patients (−3.34 ± 0.09 μV) was smaller than that evoked in the controls (−4.29 ± 0.09 μV).

##### Correlation with the PANSS

**Figure 6:**
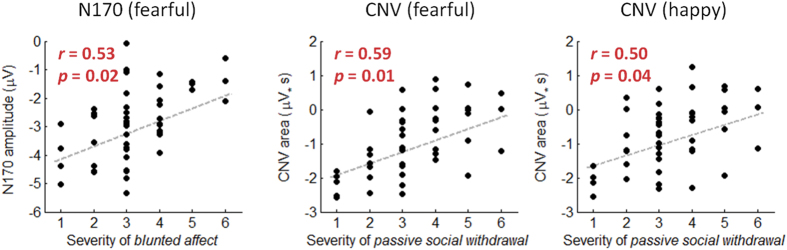
The correlation between the PANSS and the ERP measurements of patients. The values of two-tailed Pearson’s *r* correlation and associated *p* are shown in red.

According to the N170 results reported above, correlations were performed between negative items and N170 amplitude (left and right hemispheres) in the fearful condition. The severity of blunted affect was found to be correlated significantly with the N170 amplitude at the left hemisphere (*r* = 0.53; corrected *p* = 0.018; Fig. 6).

#### The CNV

##### Area amplitude

**Figure 7:**
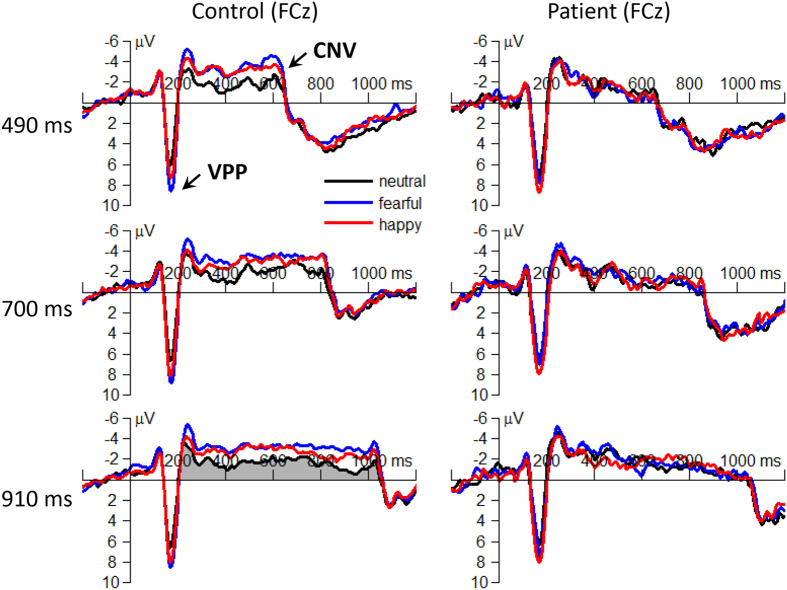
The grand-mean ERP waveforms of the VPP and the CNV components in neutral, fearful and happy conditions in the patients and controls. The CNV was estimated using an area measurement which was calculated based on the integral under the ERP waveforms between two zero crossing points on the time axis (refer to the light black region in the lower-left corner of the figure).

**Figure 8:**
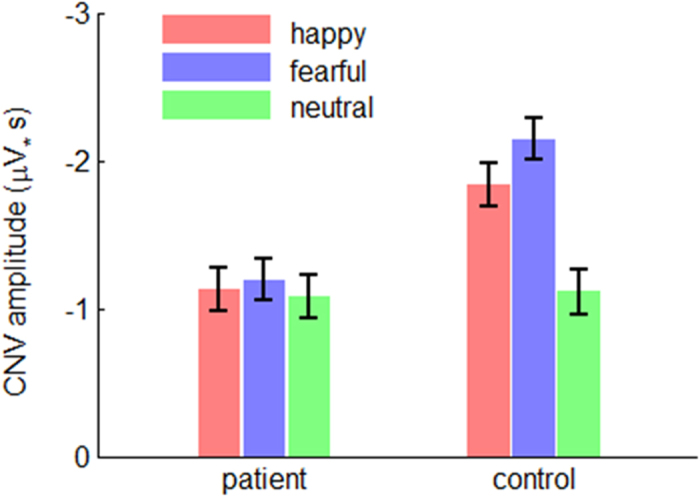
The interaction effect of emotion by group on the CNV area amplitude. Bars represent ± standard error of the mean.

The interaction effect of emotion by group was significant (*F*(2,182) = 23.0; *p* < 0.001; 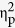 = 0.202; Figs 7 and 8). The CNV area evoked in the controls (*F*(2,184) = 57.2; *p* < 0.001) was smaller in the neutral condition (−1.11 ± 0.12 μV•s) compared with that in the happy (−1.83 ± 0.14 μV•s; *p* < 0.001) and the fearful conditions (−2.14 ± 0.12 μV•s; *p* < 0.001); and it was larger in the fearful than in the happy condition (*p* = 0.006). However, the emotion effect did not show any significant difference in the patients (*F*(2,184) < 1). Furthermore, it is found that the CNV area was larger in the controls than that in the patients both in the fearful (*F*(1,91) = 22.4; *p* < 0.001; patient = −1.20 ± 0.14 μV•s, control = −2.14 ± 0.14 μV•s) and happy conditions (*F*(1,91) = 12.1; *p* = 0.001; patient = −1.13 ± 0.14 μV•s, control = −1.83 ± 0.14 μV•s); however, the CNV did not show any significant difference between the two groups in the neutral condition (*F*(1,91) < 1; patient = −1.08 ± 0.15 μV•s, control = −1.11 ± 0.15 μV•s).

The main effect of group was significant (*F*(1,91) = 8.93; *p* = 0.004; 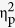 = 0.089). The CNV area evoked in the patients (−1.13 ± 0.13 μV•s) was smaller than that evoked in the controls (−1.70 ± 0.13 μV•s).

##### Correlation with the PANSS

According to the CNV results reported above, correlations were performed between negative items and the area amplitude of the CNV in the fearful/happy conditions. The severity of passive/apathetic social withdrawal was found to be correlated significantly with the 490-ms CNV area in the fearful and the happy conditions (fearful: *r* = 0.59, corrected *p* = 0.012; happy: *r* = 0.50, corrected *p* = 0.035) (Fig. 6).

## Discussion

### ERP components index impairments in different psychological procedures

The present study investigated the temporal perception of emotional faces in people with and without schizophrenia in order to better understand the nature of emotion deficits in schizophrenia. Three ERP components showed different patterns between the two groups. First, the attention-related P1 component^34^ displayed a significant group effect, with larger amplitudes in the controls than in the patients; at the same time, no interaction effect of emotion by group was found (Fig. 4). Considering that the occipital P1 can reflect early attentional modulation by emotion^35^,^36^, the current P1 result suggested that the bottom-up visual processing and its associated attention allocation were impaired for both emotional and neutral stimuli in schizophrenic patients.

Second, significant interaction effect of emotion by group was found in the N170, which is a face-sensitive ERP component and is often modulated by emotional faces^14^,^15^. Although there was also a significant group effect (larger amplitudes in the controls than in the patients), the N170 result emphasized that compared with happy and neutral faces, schizophrenic patients were more impaired in fearful face processing (Fig. 5).

Similar with the statistical result of N170, both interaction effect and main effect of group were significant on the CNV, a sensitive index for online timing^16^,^17^,^18^,^19^,^20^. The observed smaller CNV in response to fearful and happy faces in schizophrenia indicated that compared with the controls, the patients not only showed general dysfunction in temporal perception, but, more important, had a larger damage when they timing emotional, compared with neutral, stimuli (Fig. 7).

Unlike the ERP data, whose components reflected separate psychological procedures, the behavioral data only provided an overall characterization of the deficits in schizophrenia. All the three behavioral measures showed a main effect of group, indicating the patients had a general impairment when performing the task of time perception, irrespective the emotional content (fear, happiness or neutral) of stimuli. Furthermore, response data suggested that while the controls overestimated the duration of emotional (i.e. happy and fearful) faces, the patients underestimated the duration of emotional and neutral faces (Fig. 2A) and exhibited less temporal precision than the controls (Fig. 2B). In addition, the interaction effect of emotion by group on response time was significant: the shorter RT in fearful than in neutral condition for healthy participants may indicate the well-known “negativity bias” in emotion processing^37^; the longer RT in fearful than in neutral conditions for the patients was consistent with the result of N170, suggesting that schizophrenic patients were more severely impaired for fearful rather than happy facial processing.

Since the focus of the current study was the impairment in time perception of emotional stimuli, the later part of the Discussion mainly concerns the time- and emotion-related CNV and N170 components.

### Impaired emotional time perception

The present result in the healthy subjects is consistent with previous studies in non-psychotic people reporting that the duration of happy and fearful faces is always overestimated compared to neutral ones^38^,^39^. This phenomenon is usually thought to be linked to the increase of the physiological arousal level in response to emotional stimuli^11^,^40^. The increased arousal accelerates the pacemaker in the pacemaker-accumulator model^41^, leading to a greater number of accumulated pulses, thus resulting in overestimation of time^42^,^43^. (Note: the psychological mechanism of timing is usually explained by the pacemaker-accumulator model. In brief, the model includes an internal pacemaker that sends pulses to an accumulator. The longer the stimulus duration, the more pulses are accumulated, and the longer the subjective duration is judged to be). Since the accumulation of pulses is reflected by the CNV on the brain activity level, the increased arousal caused by happy and fearful faces also leads to larger CNV amplitudes^18^. This ability of unconscious time distortion under the influence of emotion facilitates people to optimize their responses to various emotional events in the environment^11^. For example, people tend to overestimate the duration of fearful pictures, which enlarges the danger delivered by the picture and prompts individuals to fight or flight^44^. Thus, the effect of emotion on time perception has an evolutionary and adaptive significance and is essential for everyday activities^40^.

Unfortunately, this emotional effect on timing totally disappeared in schizophrenic patients, as shown in our behavioral and CNV results. Here we preliminarily propose a possible mechanism underlying this impairment in schizophrenia. According to the theory of embodying emotion, individuals automatically imitate the perceived facial expressions and this imitation process produces an actual experience of the same emotion^45^. The effect of embodied emotion on time perception suggests that people synchronize activities with others, and that sharing other individuals’ time implies a desire to empathize with them^11^,^46^. Consistent with this inference, studies showed that the overestimation of the duration of angry and happy faces was not observed when the imitation of facial expressions was inhibited^39^; and that the low-empathy subjects showed a reduced temporal overestimation for angry faces in compared with the high-empathy subjects^47^. Therefore we might assume that in the current task, the empathy deficit in schizophrenia^48^ prevented emotional faces from inducing appropriate arousal level in patients, resulting in a slower pacemaker rate in them, which in turn gave rise to a temporal underestimation. However, since we mistakenly did not require the patients/controls to explicitly assess the valence and the arousal of facial expression pictures, this data interpretation needs to be proved in future studies.

An alternative interpretation for the convergent CNVs in the patients is that insufficient attention was allocated to emotional faces, due to negative symptoms such as passive/apathetic social withdrawal. It has been proposed that the supplementary motor area (SMA) is the neural substrate of the timing function reflected by the CNV^17^,^18^,^49^. Since increasing attention to timing enhances the activation of the SMA^50^, we can infer that the level of neural activation contributing to time perception (i.e. the CNV area amplitude) depends on the amount of attention paid to the target. As a result, the observed smaller CNV area in response to fearful and happy faces in schizophrenia reflected that compared with the control subjects, the patients could not keep sustained attention on emotional facial expressions.

In addition, it was also found in Fig. 6 that the CNV amplitudes increased with temporal durations. This pattern is inconsistent with some earlier CNV studies^20^,^32^, which indicated that the CNV peaks at the end of stimulus duration and then declines when the perceived stimulus is shorter than the “standard” duration (700 ms in our study); while the stimulus longer than the standard duration evokes the CNV with a peak around the end of the standard duration. This discrepancy may be due to two reasons. First, the involved cognitive systems during time estimation may be slightly different: the testing stimuli were emotional faces in this study while they were simple stimuli without emotion (e.g. the illumination of a diode) in earlier CNV studies. Second, the CNV pattern may be different across different electrodes^32^. Nevertheless, the correlation between the amplitude of the CNV and the estimated duration is still in debate^51^ and is not the focus of the current study.

### Preserved early response to positive emotion

Another interesting finding of the present study was that the early emotional processing of happy faces was preserved to a large extent when patients performing the time perception task. However, this happy *vs*. neutral difference in the N170/VPP disappeared in the later stage of emotional time perception, as reflected by the CNV.

The finding that early emotional processing of happy, rather than fearful, faces was largely preserved in schizophrenic subjects is in line with previous observations suggesting that abnormalities in negative, but not positive emotional processing may be core to affective disturbance in schizophrenia^52^,^53^. Studies of emotional processing have shown that patients are often associated with decreased activation and accuracy in responses to facial expressions of fear, anger, and disgust, compared with happy faces^54^. Furthermore, it was found that people with schizophrenia tend to show greater deficits for the recognition of negative compared with positive emotions, and of the negative emotions, patients show the greatest impairment for the perception of fear^55^,^56^.

Taken together, the results of N170 and CNV suggested that patients may adequately perceive and experience the emotion of happy in the moment, but their temporal judgment of happy faces is largely impaired. Consistently, as we mentioned in the introduction, many studies in schizophrenia showed that patients and controls usually report experiencing similar levels of pleasure in response to a pleasant mood induction^3^,^57^; the patients, however, have an anticipatory pleasure deficit and do not expect to experience positive affect when engaging in future goal-directed activities^3^,^9^,^58^. As a complementary study to the previous anticipatory investigations, the current work directly examined the timing function for emotional stimuli in schizophrenia. Our results further underlined the importance of taking time dimension into consideration when examining the emotional deficits in schizophrenia.

### Correlation between ERPs and emotional/social impairments

One of the important contributions of this study was that we presented a preliminary argument for the need to understand the relationships between blunted affect, passive social withdrawal, and the function of emotional time perception. It was found that the N170 amplitude in the fearful condition was correlated with the severity of blunted affect, the latter of which has been suggested as the most important negative symptom associated with emotion deficits and could independently predict the performance in emotion-related tasks^59^. In addition, the CNV in the happy and the fearful conditions was correlated with the severity of passive/apathetic social withdrawal, the latter of which is a primary negative symptom that assesses diminished interest and initiative in social interactions due to passivity or apathy^60^.

Our result highlights the potential value of ERP measures as direct predictors of emotional/social impairments in patients. Furthermore, as numerous studies have documented the difficulties of improving negative symptoms^61^,^62^, this study presents emotional time perception as a promising target for treatment efforts and suggests that remediation of emotional timing deficits may results in improved functional outcome.

## Conclusion

The current study examined the interaction of emotion and time perception in schizophrenia. Using neutral faces to provide a putative baseline, we demonstrated in the patients that the emotional processing and timing for facial expressions might have a pattern of two-stage deterioration: while the early brain response to happy, but not fearful, faces was largely preserved, the function of emotional timing was drastically impaired for both fearful and happy faces. Findings from the present work point to the importance of considering the time dimension of emotional processing in schizophrenia, based on which we are likely to discover aspects of emotional deficits that would be unnoticed in other studies. It was observed that schizophrenic patients were not only less accurate in time estimation, but also showed a particular kind of perception deviation in emotional timing (underestimation compared with healthy subjects). In light of the result, we suggest that the direction and magnitude of the temporal deviation for emotional stimuli may provide novel insights into the core pathophysiology and offer quantitative biomarkers for specific emotional/social dysfunctions in schizophrenia.

## Additional Information

**How to cite this article**: Zhang, D. *et al*. Perception of the duration of emotional faces in schizophrenic patients. *Sci. Rep*. **6**, 22280; doi: 10.1038/srep22280 (2016).

## Supplementary Material

Supplementary Information

## Figures and Tables

**Table 1:** Demographic and clinical data of patient and control groups. Descriptive data are presented as mean (range).

Characteristics	Patient (n = 47)	Control (n = 46)	Statistics
Mean age, y	31.3 (18–42)	33.6 (21–56)	*t*(91) = −1.54, *p* = 0.127
Education time, y	13.4 (9–16)	14.2 (9–16)	*t*(91) = −1.78, *p* = 0.078
IQ^a^	100 (75–127)	103 (77–129)	*t*(91) = −1.47, *p* = 0.145
Sex, male/female	27/20	26/20	χ^2^(1) = 0.008, *p* = 0.928
Handedness, right/left	47/0	46/0	
Length of illness, m	140.8 (2–300)		
Age at disease onset, y	19.6 (14–38)		
Neuroleptic, typical/atypical/both	0/44/3		
Subtype, paranoid/undifferentiated	18/29		
Chlorpromazine equivalents, mg/d^b^	584.7 (268–948)		
PANSS score			
Positive scale	15.1 (7–34)		
Negative scale	16.2 (10–30)		
General Psychopathology scale	31.3 (21–50)		

^a^The Wechsler Adult Intelligence Scale (fourth edition)^63^,^64^,^65^.

^b^According to the reference of Woods^66^.
