# Supplementary material for: Perception of the duration of emotional faces in schizophrenic patients
Source: Sci Rep. 2016 Feb 29;6:22280. doi: 10.1038/srep22280 (PMC4770274; doi:10.1038/srep22280)
Supplement: Supplementary Information [file srep22280-s1.pdf]

Perception of the duration of emotional faces in schizophrenic patients

Dandan Zhang[1,#], Yanli Zhao [2,#], Yunzhe Liu [1], Shuping Tan [2,*]

1 Institute of Affective and Social Neuroscience, Shenzhen University, Shenzhen, 518060, China
2 Center for Psychiatric Research, Beijing Huilongguan Hospital, Beijing, 100096, China

* Corresponding author. Beijing Huilongguan Hospital, Beijing 100096, China. Tel.: + 86 10 62716905; fax: + 86 10 62718210. E-mail address: shupingtan@126.com.

# Both authors contributed equally to the study

The Authors have declared that there are no conflicts of interest in relation to the subject of this study.

Additional results

Behaviors

Accuracy rate

The interaction effect of stimulus duration by group was significant ($F(2,182) = 8.94$; $p < 0.001$; $\eta_p^2 = 0.089$). The accuracy rate (ACC) in the patients ($F(2,182) = 9.61$; $p < 0.001$) was higher in the 490-ms condition (0.596 ± 0.032) compared with the 700-ms condition (0.407 ± 0.040; $p = 0.002$) and the 910-ms condition (0.420 ± 0.032; $p = 0.002$). However, the stimulus duration effect showed a different pattern in the controls ($F(2,182) = 14.8$; $p < 0.001$); the ACC was lower in the 700-ms condition (0.511 ± 0.032) compared with the 490-ms condition (0.689 ± 0.015; $p < 0.001$) and the 910-ms condition (0.769 ± 0.027; $p < 0.001$). In addition, the interaction effect of emotion by stimulus duration was significant ($F(4,364) = 6.11$; $p < 0.001$; $\eta_p^2 = 0.063$).

The main effect of emotion was significant ($F(2,182) = 3.98$; $p = 0.020$; $\eta_p^2 = 0.042$). The ACC was higher in the fearful condition (0.576 ± 0.009) than in the neutral condition (0.553 ± 0.010).

The main effect of stimulus duration was significant ($F(2,182) = 15.5$; $p < 0.001$; $\eta_p^2 = 0.146$). The ACC was lower in the 700-ms condition (0.459 ± 0.026) compared with that in the 490-ms condition (0.642 ± 0.018; $p < 0.001$) and in the 910-ms condition (0.595 ± 0.021; $p = 0.002$).

The "short", "equal", and "long" responses

The interaction effect of response by group was significant ($F(2,182) = 13.7$; $p < 0.001$; $\eta_p^2 = 0.131$). The patients ($F(2,182) = 12.0$; $p < 0.001$) gave "short" responses (19.9 ± 0.82 counts) more frequently than "long" responses (11.0 ± 1.03 counts; $p < 0.001$). However, the controls ($F(2,182)$

= 3.30; p = 0.039) gave "long" responses (19.0 ± 1.05 counts) more frequently than "short" responses (14.5 ± 0.82 counts; $p < 0.001$).

The interaction effect of stimulus duration by response by group was significant ($F(4,364)$ = 39.1; $p < 0.001$; η_p^2 = 0.301) (Figure 2B). Compared with the controls, the patients gave more "short" responses ($F(1,91)$ = 30.0; $p < 0.001$; patient = 17.6 ± 1.04 counts; control = 9.50 ± 1.05 counts) and less "long" responses ($F(1,91)$ = 5.41; p = 0.022; patient = 10.1 ± 1.26 counts; control = 14.3 ± 1.28 counts) in the 700-ms condition. Compared with the controls, the patients gave more "short" responses ($F(1,91)$ = 123; $p < 0.001$; patient = 13.2 ± 0.72 counts; control = 1.78 ± 0.73 counts) and less "long" responses ($F(1,91)$ = 75.4; $p < 0.001$; patient = 19.0 ± 1.39 counts; control = 36.2 ± 1.41 counts) in the 910-ms condition. However, the similar response pattern was not found in the 490-ms condition.

The interaction effect of emotion by response was significant ($F(4,364)$ = 8.96; $p < 0.001$; η_p^2 = 0.090). The interaction effect of stimulus duration by response was significant ($F(4,364)$ = 313; $p < 0.001$; η_p^2 = 0.775). The interaction effect of emotion by stimulus duration was significant ($F(4,364)$ = 3.61; p = 0.007; η_p^2 = 0.038). The interaction effect of emotion by stimulus duration by response was significant ($F(8,728)$ = 2.99; p = 0.009; η_p^2 = 0.032).

The main effect of emotion was significant ($F(2,182)$ = 6.62; p = 0.002; η_p^2 = 0.068). The valid responses given to fearful faces (16.2 ± 0.08 counts) were larger compared to those given to happy (16.0 ± 0.08 counts; p = 0.049) and neutral faces (15.8 ± 0.09 counts; p = 0.001).

Response time

The interaction effect of stimulus duration by group was significant ($F(2,182)$ = 8.73; $p < 0.001$;

η_p^2 = 0.088). The response time (RT) in the patients ($F(2,184) = 29.2$; $p < 0.001$) decreased with increasing stimulus duration (786 ± 14.9 ms, 715 ± 14.4 ms, 652 ± 12.8 ms in the 490-, the 700-, and the 910-ms conditions; *p*s < 0.001). This effect of stimulus duration on the RT was more significant in the controls ($F(2,184) = 68.5$; $p < 0.001$; 697 ± 9.5 ms, 657 ± 9.2 ms, 525 ± 14.7 ms in the 490-, the 700-, and the 910-ms conditions; *p*s < 0.001).

The interaction effect of emotion by stimulus duration was significant ($F(4,364) = 5.06$; $p = 0.001$; $\eta_p^2 = 0.053$).

The main effect of stimulus duration was significant ($F(2,182) = 174$; $p < 0.001$; $\eta_p^2 = 0.657$). Post-hoc pairwise comparisons indicated that the RT measures in each pair of duration conditions showed significant differences (*p*s < 0.001); the RT decreased with increasing stimulus duration (742 ± 8.9 ms, 686 ± 8.6 ms, 589 ± 9.7 ms in the 490-, the 700-, and the 910-ms conditions).

ERPs

The P1

Peak amplitude

The interaction effect of hemisphere by group was significant ($F(1,91) = 5.75$; $p = 0.019$; $\eta_p^2 = 0.059$) (Figure 4). The P1 amplitude evoked in the controls had a right hemisphere advantage ($F(1,91) = 17.0$; $p < 0.001$; left = 3.20 ± 0.12 μV, right = 3.77 ± 0.12 μV) while the hemisphere effect was not significant in the patients ($F(1,91) < 1$).

The main effect of hemisphere was significant ($F(1,91) = 12.0$; $p = 0.001$; $\eta_p^2 = 0.116$). The P1 amplitude in the left hemisphere (2.74 ± 0.08 μV) was smaller than that in the right hemisphere (3.08 ± 0.08 μV).

No interaction effect of emotion by group was found (Figure 4).

Peak latency

The main effect of group was significant ($F(1,91) = 58.7$; $p < 0.001$; $\eta_p^2 = 0.392$). The P1 latency evoked in the patients (119 ± 1.39 ms) was shorter than that evoked in the controls (134 ± 1.40 ms).

The main effect of hemisphere was significant ($F(1,91) = 4.58$; $p = 0.035$; $\eta_p^2 = 0.048$). The P1 latency was longer in the left hemisphere (129 ± 1.32 ms) compared with that in the right hemisphere (125 ± 1.33 ms).

The N170

Peak amplitude

The main effect of emotion was significant ($F(2,182) = 14.6$; $p < 0.001$; $\eta_p^2 = 0.139$). The N170 amplitude was smaller in response to neutral faces (-3.54 ± 0.09 μV) compared with that in the happy condition (-4.07 ± 0.09 μV; $p < 0.001$) and in the fearful condition (-3.84 ± 0.08 μV; $p = 0.005$).

The main effect of hemisphere was significant ($F(1,91) = 42.3$; $p < 0.001$; $\eta_p^2 = 0.318$). The N170 amplitude in the left hemisphere (-3.45 ± 0.08 μV) was smaller than that in the right hemisphere (-4.18 ± 0.09 μV).

Peak latency

The main effect of group was significant ($F(1,91) = 44.1$; $p < 0.001$; $\eta_p^2 = 0.326$). The N170

latency evoked in the patients (174 ± 1.05 ms) was shorter than that evoked in the controls (184 ± 1.06 ms).

The VPP

The statistical results of the VPP were very similar with those of the N170.

Peak amplitude

The interaction effect of emotion by group was significant ($F(2,182) = 42.9$; $p < 0.001$; $\eta_p^2 = 0.321$) (Figure 6). The VPP amplitude evoked in the patients ($F(2,182) = 48.7$; $p < 0.001$) was larger in the happy condition (7.88 ± 0.15 μV) compared with that in the fearful condition (6.57 ± 0.15 μV; $p < 0.001$) and in the neutral condition (6.13 ± 0.16 μV; $p < 0.001$). However, the emotion effect showed a different pattern in the controls ($F(2,182) = 91.5$; $p < 0.001$): the VPP amplitude was smaller in the neutral condition (8.28 ± 0.16 μV) compared with that in the happy condition (9.91 ± 0.15 μV; $p < 0.001$) and in the fearful condition (10.8 ± 0.17 μV; $p < 0.001$); and the VPP amplitude was larger in the fearful condition compared with that in the happy condition ($p < 0.001$).

The main effect of emotion was significant ($F(2,182) = 97.8$; $p < 0.001$; $\eta_p^2 = 0.518$). The VPP amplitude was smaller in response to neutral faces (7.20 ± 0.11 μV) compared with that in the happy condition (8.90 ± 0.11 μV; $p < 0.001$) and in the fearful condition (8.67 ± 0.11 μV; $p < 0.001$).

The main effect of group was significant ($F(1,91) = 312$; $p < 0.001$; $\eta_p^2 = 0.774$). The VPP amplitude evoked in the patients (6.86 ± 0.11 μV) was smaller than that evoked in the controls

(9.65 ± 0.11 μV).

Peak latency

The main effect of group was significant (F(1,91) = 7.09; p = 0.009; η_p^2 = 0.072). The VPP latency evoked in the patients (171 ± 1.58 ms) was shorter than that evoked in the controls (177 ± 1.60 ms).

The CNV

Area amplitude

The main effect of emotion was significant (F(2,182) = 35.4; p < 0.001; η_p^2 = 0.280). The CNV area was smaller in response to neutral faces (-1.09 ± 0.11 μV•s) compared with that in the happy condition (-1.48 ± 0.10 μV•s; p < 0.001) and in the fearful condition (-1.67 ± 0.10 μV•s; p < 0.001); and it was larger in response to fearful than to happy faces (p = 0.018).

The main effect of stimulus duration was significant (F(2,182) = 42.4; p < 0.001; η_p^2 = 0.318). The CNV area increased along with the stimulus duration (the 490-ms condition = -1.00 ± 0.08 μV•s, the 700-ms condition = -1.37 ± 0.11 μV•s, the 910-ms condition = -1.86 ± 0.13 μV•s; pairwise comparisons: ps < 0.010).

Other relative factors

Diagnostic subtype

To examine the emotion-modulated time perception in patients with different schizophrenia subtypes, the behavioral/ERP data for paranoid schizophrenia, undifferentiated schizophrenia and

healthy controls were analyzed comparatively. There were no significant differences across the three groups with respect to age ($F(2,90) = 1.68$; $p = 0.192$; $\eta_p^2 = 0.036$), duration of education ($F(2,90) < 1$; $\eta_p^2 = 0.012$) and IQ ($F(2,90) = 1.54$; $p = 0.221$; $\eta_p^2 = 0.033$).

The RT data showed a significant main effect of group ($F(2,90) = 13.2$; $p < 0.001$; $\eta_p^2 = 0.227$). The RT was longer in the undifferentiated patients (734 ± 14.5 ms) compared with the controls (639 ± 11.5 ms, $p < 0.001$) and the paranoid patients (660 ± 18.4 ms, $p = 0.007$), whereas there was no difference between the paranoid patients and the controls ($p = 0.998$). The interaction effect of emotion by group was significant ($F(4,180) = 5.48$; $p < 0.001$; $\eta_p^2 = 0.109$). The RT in the undifferentiated patients ($F(2,180) = 6.85$; $p = 0.001$) was longer in the fearful condition (757 ± 17.1 ms) compared with that in the neutral condition (698 ± 18.0 ms, $p = 0.002$) while the RT in the controls and in the paranoid patients did not show any significant difference among emotional conditions ($F(2,180) < 1$).

The CNV area showed a significant main effect of group ($F(2,90) = 9.41$; $p < 0.001$; $\eta_p^2 = 0.173$). The CNV area was smaller in the undifferentiated patients (-0.83 ± 0.16 μV•s) compared with the controls (-1.70 ± 0.13 μV•s, $p < 0.001$) and the paranoid patients (-1.62 ± 0.20 μV•s, $p = 0.010$) whereas there was no difference between the paranoid patients and the controls ($p = 1.000$).

Medications, age of illness onset and duration of illness

Two-tailed Pearson's *r* correlation was performed to explore the effects of medications (i.e., chlorpromazine equivalents), age of illness onset and duration of illness on the behavioral/ERP measurements of the patients. No significance was found after the correction for multiple comparisons.
